# Small Bowel Obstruction in a Virgin Abdomen: A Case Report

**DOI:** 10.7759/cureus.4594

**Published:** 2019-05-03

**Authors:** Mohamed Ahmed, Saba Habis, Rasha Saeed, Ahmed Mahmoud, Naseem Attar

**Affiliations:** 1 Surgery, University of California, Riverside, USA; 2 Internal Medicine, Riverside Community Hospital, Riverside, USA; 3 Surgery, Arrowhead Regional Medical Center, Fontana, USA; 4 Surgery, Riverside Community Hospital, Riverside, USA

**Keywords:** small bowel obstruction, foreign body

## Abstract

Small bowel obstruction (SBO) is a major cause of morbidity and financial burden in hospitals around the world. Foreign body (FB) ingestion as a cause is rare. While most cases are straight forward, some can be extremely subtle. We present a case of SBO caused by an undigested piece of pineapple core.

## Introduction

Patients presentation after foreign body ingestion is usually straightforward and 80% of ingested foreign bodies reaching the stomach will pass spontaneously [1]. Surgery should be reserved for those who have an acute abdomen [2]. We present a case of small bowel obstruction secondary to undigested pineapple core.

## Case presentation

**Figure 1:**
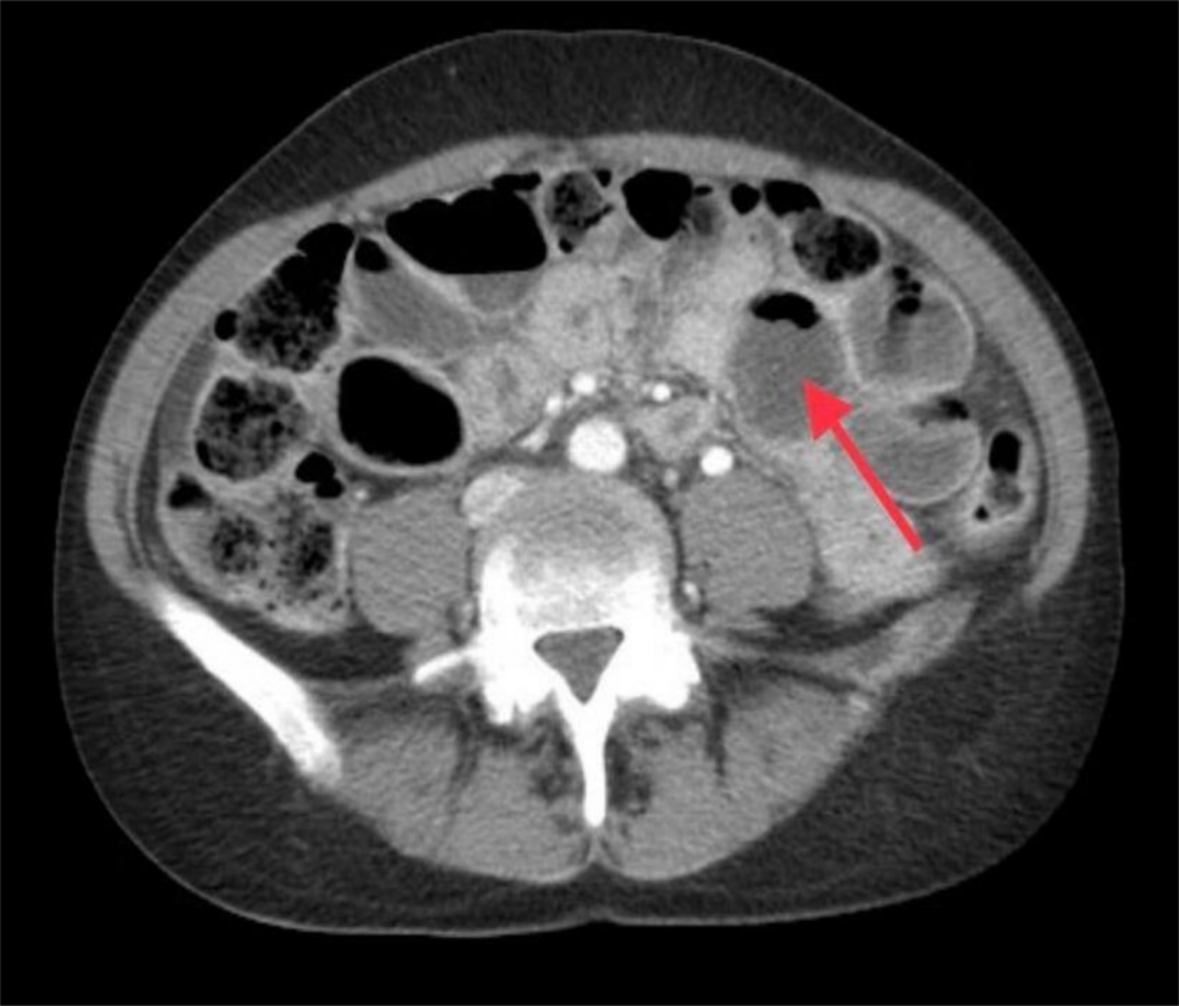
CT scan abdomen. Consistent with small bowel obstruction. Distended small bowel loop (Red arrow). CT: Computed tomography

A 52-year-old female with no past medical or surgical history presented to our emergency room with two days history of abdominal pain, nausea, vomiting and obstipation. The patient had a distended abdomen, discomfort on deep palpation, with no peritoneal signs. Her white blood cell count was mildly elevated and low potassium was replaced. Computed tomography was concerning for small bowel obstruction (Figure 1).

**Figure 2:**
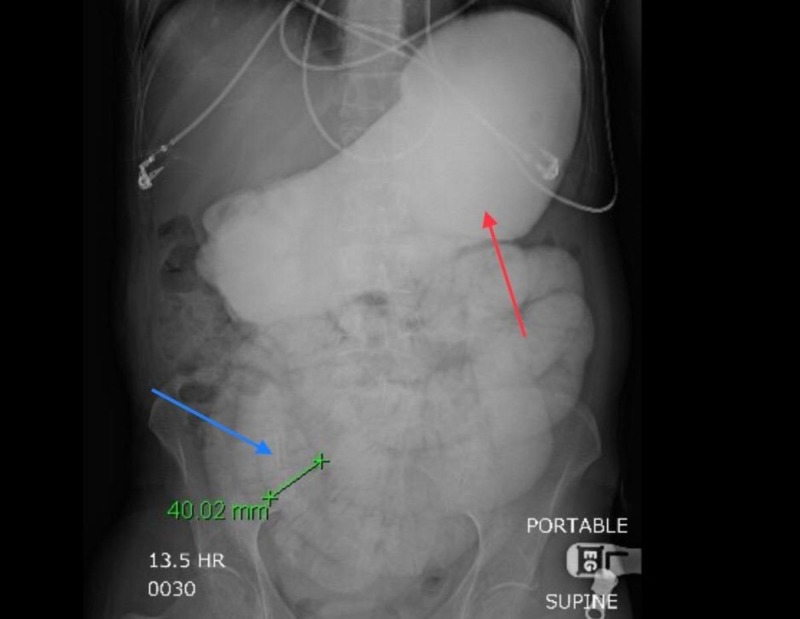
Small bowel follow-through. Distended stomach (Red Arrow). Distended small bowel (Blue arrow).

The patient was admitted to the hospital and small bowel follow-through the following day revealed moderate distention of the stomach, multiple distended small bowel loops and no evidence of contrast in the cecum at 14 hours consistent with small bowel obstruction (Figure 2).

**Figure 3:**
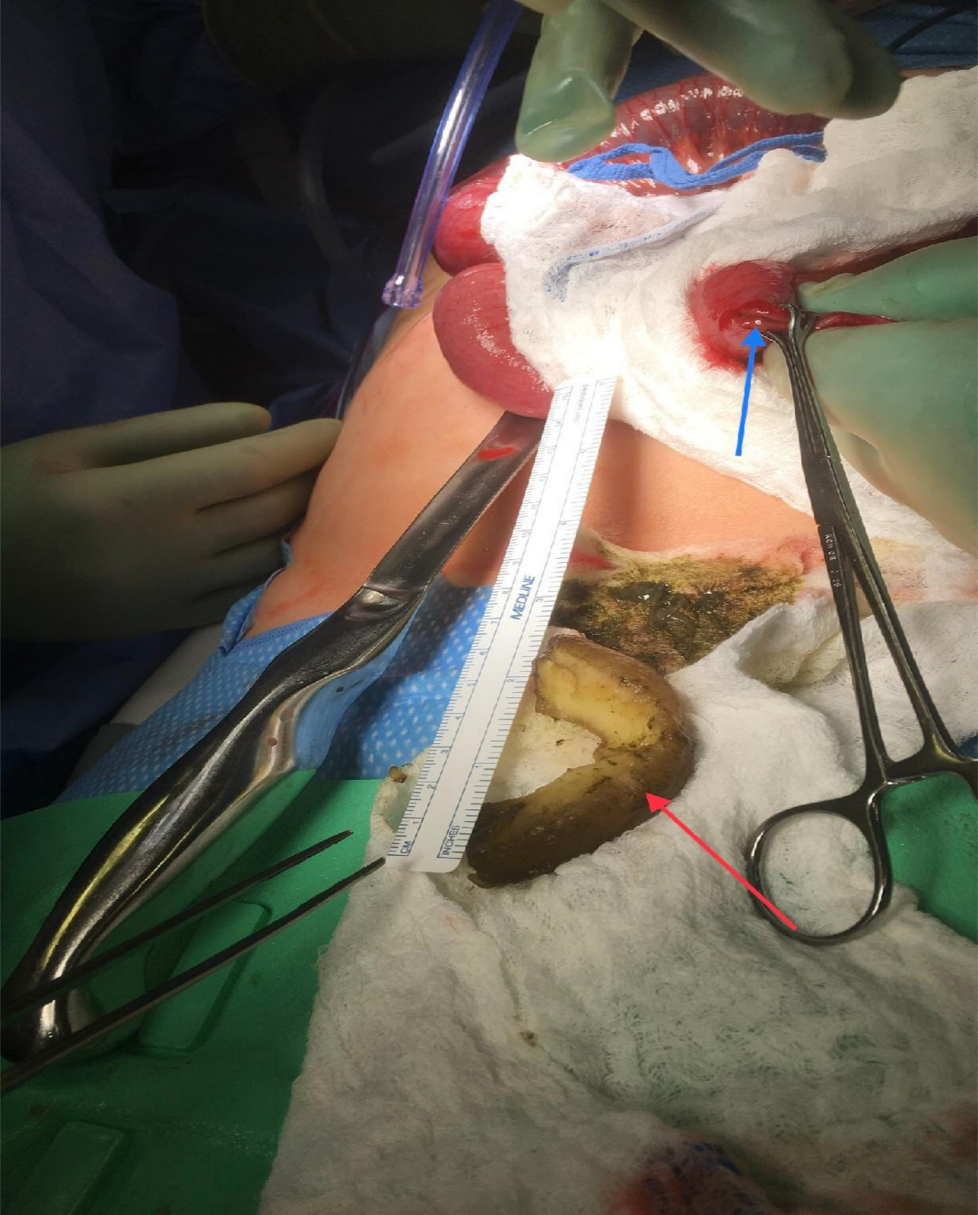
Exploratory laparotomy. Enterotomy (Blue Arrow). Pineapple core (Red Arrow).

The patient was taken to the operating room, and exploratory laparotomy with retrieval of a foreign body via an enterotomy was performed (Figure 3).

The patient recalled ingesting pineapple core as a source of fiber the day prior to her symptoms. She did well and was discharged from the hospital.

## Discussion

Small bowel obstruction (SBO) etiology in developed countries includes adhesions (74%), Crohn's disease (7%), neoplasia (5%), hernia (2%), radiation (1%), and miscellaneous (11%) [3]. In contrast, developing countries etiology includes adhesions (34%), hernia (16%), malignancy (13.5%) and tuberculous stricture (10%) [4]. In the pediatric population foreign body ingestion is a known cause of abdominal pain, most of which will pass spontaneously and less than 1% will require surgical removal [5,6]. Acute intestinal obstruction due to foreign bodies is rare in adults [7]. Small bowel foreign bodies can cause obstruction, perforation and bleeding [8]. Most patients can be managed conservatively or with endoscopic retrieval and only a small minority will require surgery [9]. Pineapple core known to have high fiber content led to intestinal obstruction in our patient.

## Conclusions

Patients' presentation after foreign body ingestion is usually straightforward but on occasions can be extremely subtle. Pineapple core is a good source of fiber, however, the ingestion of large undigested pieces led to an intestinal obstruction in our patient.
